# Recessive nephrocerebellar syndrome on the Galloway-Mowat syndrome spectrum is caused by homozygous protein-truncating mutations of *WDR73*

**DOI:** 10.1093/brain/awv153

**Published:** 2015-06-12

**Authors:** Robert N. Jinks, Erik G. Puffenberger, Emma Baple, Brian Harding, Peter Crino, Agnes B. Fogo, Olivia Wenger, Baozhong Xin, Alanna E. Koehler, Madeleine H. McGlincy, Margaret M. Provencher, Jeffrey D. Smith, Linh Tran, Saeed Al Turki, Barry A. Chioza, Harold Cross, Gaurav V. Harlalka, Matthew E. Hurles, Reza Maroofian, Adam D. Heaps, Mary C. Morton, Lisa Stempak, Friedhelm Hildebrandt, Carolin E. Sadowski, Joshua Zaritsky, Kenneth Campellone, D. Holmes Morton, Heng Wang, Andrew Crosby, Kevin A. Strauss

**Affiliations:** 1 Department of Biology and Biological Foundations of Behaviour Program, Franklin and Marshall College, Lancaster, PA 17604, USA; 2 Clinic for Special Children, Strasburg, PA 17579, USA; 3 RILD Wellcome Wolfson Centre, Royal Devon and Exeter NHS Foundation Trust, Barrack Road, Exeter, EX2 5DW, UK; 4 Human Genetics and Genomic Medicine, Faculty of Medicine, University of Southampton, UK; 5 Wessex Clinical Genetics Service, Princess Anne Hospital, Southampton, UK; 6 Department of Pathology and Laboratory Medicine, Children’s Hospital of Philadelphia and Perelman School of Medicine, University of Pennsylvania, Philadelphia, PA 19104, USA; 7 Shriners Hospital Paediatric Research Centre, Temple University School of Medicine, Philadelphia, PA 19140, USA; 8 Division of Renal Pathology, Vanderbilt University School of Medicine, Nashville, TN 37232, USA; 9 New Leaf Clinic for Special Children, Mount Eaton, OH 44659, USA; 10 Department of Paediatrics, Akron Children’s Hospital, Akron, OH 44302, USA; 11 DDC Clinic for Special Needs Children, Middlefield, OH 44062, USA; 12 Wellcome Trust Sanger Institute, Hinxton, Cambridge CB10 1SA, UK; 13 Medical Research, RILD Wellcome Wolfson Centre, University of Exeter Medical School, Exeter EX1 2LU, UK; 14 Department of Ophthalmology, University of Arizona College of Medicine, Tucson, AZ 85711, USA; 15 Department of Pathology, University Hospitals Case Medical Centre, Cleveland, OH 44106, USA; 16 Case Western Reserve University School of Medicine, Cleveland, OH 44106, USA; 17 Howard Hughes Medical Institute, Chevy Chase, MD 20815, USA; 18 Division of Nephrology, Department of Medicine, Boston Children’s Hospital, Harvard Medical School, Boston, MA 02115, USA; 19 Department of Paediatrics, Nemours/Alfred I. DuPont Hospital for Children, Wilmington, DE 19803, USA; 20 Department of Molecular and Cell Biology and Institute for Systems Genomics, University of Connecticut, Storrs, CT 06269, USA; 21 Lancaster General Hospital, Lancaster, PA 17602, USA; 22 Department of Paediatrics, Rainbow Babies and Children’s Hospital and Department of Molecular Cardiology, Cleveland Clinic, Cleveland, OH 44195, USA

**Keywords:** progressive microcephaly, nephrosis, cerebellar hypoplasia, mitosis, mTOR

## Abstract

We describe a novel nephrocerebellar syndrome on the Galloway-Mowat syndrome spectrum among 30 children (ages 1.0 to 28 years) from diverse Amish demes. Children with nephrocerebellar syndrome had progressive microcephaly, visual impairment, stagnant psychomotor development, abnormal extrapyramidal movements and nephrosis. Fourteen died between ages 2.7 and 28 years, typically from renal failure. Post-mortem studies revealed (i) micrencephaly without polymicrogyria or heterotopia; (ii) atrophic cerebellar hemispheres with stunted folia, profound granule cell depletion, Bergmann gliosis, and signs of Purkinje cell deafferentation; (iii) selective striatal cholinergic interneuron loss; and (iv) optic atrophy with delamination of the lateral geniculate nuclei. Renal tissue showed focal and segmental glomerulosclerosis and extensive effacement and microvillus transformation of podocyte foot processes. Nephrocerebellar syndrome mapped to 700 kb on chromosome 15, which contained a single novel homozygous frameshift variant (*WDR73* c.888delT; p.Phe296Leufs*26). WDR73 protein is expressed in human cerebral cortex, hippocampus, and cultured embryonic kidney cells. It is concentrated at mitotic microtubules and interacts with α-, β-, and γ-tubulin, heat shock proteins 70 and 90 (HSP-70; HSP-90), and the carbamoyl phosphate synthetase 2/aspartate transcarbamylase/dihydroorotase multi-enzyme complex. Recombinant WDR73 p.Phe296Leufs*26 and p.Arg256Profs*18 proteins are truncated, unstable, and show increased interaction with α- and β-tubulin and HSP-70/HSP-90. Fibroblasts from patients homozygous for WDR73 p.Phe296Leufs*26 proliferate poorly in primary culture and senesce early. Our data suggest that in humans, WDR73 interacts with mitotic microtubules to regulate cell cycle progression, proliferation and survival in brain and kidney. We extend the Galloway-Mowat syndrome spectrum with the first description of diencephalic and striatal neuropathology.

## Abstract (precis)

Galloway-Mowat syndrome (GMS) is a neurodevelopmental disorder characterized by microcephaly, cerebellar hypoplasia, nephrosis, and profound intellectual disability. Jinks *et al.* extend the GMS spectrum by identifying a novel nephrocerebellar syndrome with selective striatal cholinergic interneuron loss and complete lateral geniculate nucleus delamination, caused by a frameshift mutation in *WDR73*.

## Graphical abstract

**Figure:**
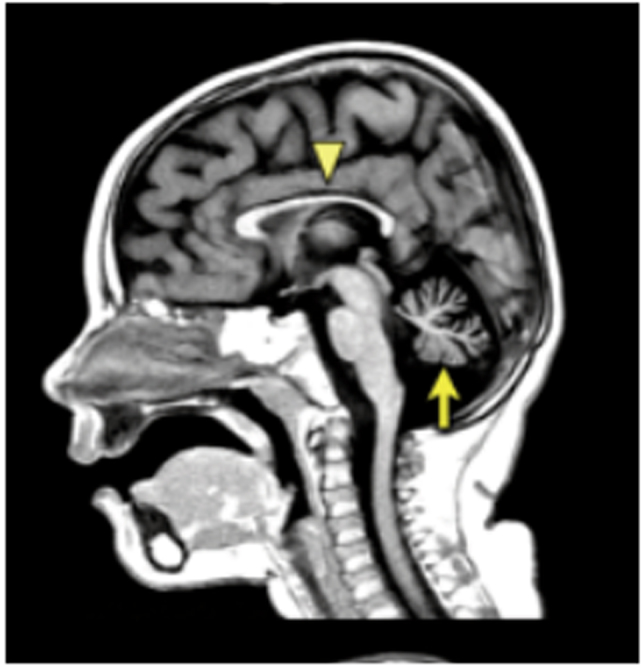
Galloway-Mowat syndrome (GMS) is a neurodevelopmental disorder characterized by microcephaly, cerebellar hypoplasia, nephrosis, and profound intellectual disability. Jinks *et al.* extend the GMS spectrum by identifying a novel nephrocerebellar syndrome with selective striatal cholinergic interneuron loss and complete lateral geniculate nucleus delamination, caused by a frameshift mutation in *WDR73*.

## Introduction

We identified 27 children from six different North American Amish demes who exhibited a nephrocerebellar syndrome (NCS) on the Galloway-Mowat spectrum (OMIM 251300) (Galloway and Mowat, 1968; Cohen and Turner, 1994; Sano *et al.*, 1995; Meyers *et al.*, 1999; Steiss *et al.*, 2005; Keith *et al.*, 2011; Ekstrand *et al.*, 2012; Colin *et al.*, 2014) characterized by progressive microcephaly, cortical visual impairment with congenital roving nystagmus, arrested psychomotor development, an extrapyramidal movement disorder, and steroid non-responsive progressive renal failure. Autozygosity mapping identified a 700 kb homozygous haplotype block on chromosome 15 shared among affected children, which we queried with exome sequencing. A single novel protein-truncating frameshift variant (*WDR73* c.888delT; p.Phe296Leufs*26) was homozygous in all affected children and heterozygous in parents. An additional 800 children with inherited nephrotic syndrome of unknown genetic cause were then screened with microfluidic multiplex PCR (Fluidigm) and next-generation sequencing (Halbritter *et al.*, 2012; Sadowski *et al.*, 2015) (Boston Children’s Hospital), and a second homozygous frameshift variant (*WDR73* c.766dupC; p.Arg256Profs*18) was identified in a child with NCS from Bulgaria. The latter variant was also recently associated with NCS on the Galloway-Mowat syndrome spectrum (characterized as ‘late-onset Galloway-Mowat syndrome’) in an unrelated Turkish child (Colin *et al.*, 2014).

Colin *et al.* (2014) associated two recessive loss-of-function mutations in *WDR73* (c.129T>G; p.Tyr43* and c.766dupC) with late-onset Galloway-Mowat syndrome in three affected children and provided a detailed description of the associated kidney pathology. WDR73 is a WD repeat (WDR) protein that is expressed in the embryonic brain and kidneys and associates with the mitotic microtubules during cell division (Colin *et al.*, 2014). WDR proteins participate in diverse cellular functions, including cell division, signal transduction, vesicle trafficking, cytoskeletal dynamics, DNA excision repair, nuclear envelope transport, and autophagy (van Nocker and Ludwig, 2003; Huebner *et al.*, 2004; Meyer, 2007; Storr *et al.*, 2009; Haack *et al.*, 2012; Saijo, 2013; Colin *et al.*, 2014), and several have been linked to developmental disorders (e.g. WDR45, WDR62, WDR81, PAFAH1B1, ERCC8 and AAAS) (Supplementary Table 1) (Gambello *et al.*, 2003; Huebner *et al.*, 2004; Bilguvar *et al.*, 2010; Gulsuner *et al.*, 2011; Haack *et al.*, 2012; Baez *et al.*, 2013).

Here we associate a novel protein-truncating mutation in *WDR73* with a nephrocerebellar syndrome on the Galloway-Mowat syndrome spectrum (herein referred to as NCS) characterized by abnormal cerebral cortical growth without polymicrogyria or heterotopia, cerebellar hypoplasia with granule layer aplasia, aberrant visual pathway development, depletion of striatal cholinergic interneurons, and focal segmental glomerulosclerosis.

Our studies of the WDR73 interactome demonstrate that WDR73 interacts with proteins vital to cell cycle and survival, including α-*(TUBA1B)*, β-*(TUBB4B)*, and γ-*(TUBG1)* tubulin, HSP-70 (*HSPA1A/HSPA1B*), HSP-90 *(HSP90AA1)*, p70S6 kinase *(RPS6KB1)*, and CAD (*CAD*; carbamoyl-phosphate synthetase 2, aspartate transcarbamylase, and dihydroorotase), a mTORC1-regulated multi-enzyme complex that mediates *de novo* pyrimidine synthesis (Ben-Sahra *et al.*, 2013; Robitaille *et al.*, 2013). *In vitro*, recombinant pathogenic WDR73 variants have increased interactions with α- and β-tubulin and HSP-90. Fibroblasts from affected children have a cell cycle disruption that substantially slows division, alters the interphase microtubule network and cell morphology, and accelerates senescence. The cell cycle defect can be rescued with wild-type WDR73. Our detailed neuropathological analysis differentiates WDR73-associated NCS from other forms of Galloway-Mowat syndrome and extends the clinical pathological spectrum. Our observations together with those of Colin *et al.* (2014) support a critical role for WDR73 in the proliferation and survival of human brain and kidney cells.

## Materials and methods

### Patients

The study was approved by Institutional Review Boards of Lancaster General Hospital, Boston Children’s Hospital, the University of Arizona, the University of Exeter, the DDC Clinic for Special Needs Children, and the Broad Institute. Parents consented on behalf of their children. Thirty patients with NCS (mean age 9.2 years, range 1.4 to 28 years) originated from three different endogamous Old Order Amish demes and hailed from six states: Pennsylvania, Ohio, Indiana, Michigan, Colorado and Montana. A Bulgarian child with NCS [5 years old at time of diagnosis and unrelated to the *WDR73* c.766dupC patient described by Colin *et al.* (2014)] was added to the study through additional screening at Boston Children’s Hospital (see below).

### Genetic mapping

Genetic mapping was conducted as previously described (Puffenberger *et al.*, 2004). Genotyping was performed using GeneChip_®_ Mapping 10K and 50K Assay Kits (Affymetrix) and single nucleotide polymorphism (SNP) data were analysed using Microsoft Excel. Genotype data came from Affymetrix GeneChip® Human Mapping 10K Xba 142 and 50K Xba Arrays and SNP positions came from Affymetrix genome annotation files. Genotype data from multiple affected individuals were examined for shared blocks of homozygosity assuming mutation and locus homogeneity. SNP data from 100 healthy Old Order Amish females were used to estimate population-specific allele frequencies. Two-point LOD scores [logarithm (base 10) of odds] were calculated for each SNP using an approach similar to Broman and Weber (1999). For each block of shared homozygous SNPs, we calculated a cumulative two-point LOD score (‘location score’) (Broman and Weber, 1999) that indicated the relative probability that the disease gene resided in the homozygous block.

### Exome sequencing

Exome sequencing was performed at the Broad Institute using the Agilent SureSelect All Exon Kit (v.1, 38 Mb) as previously described (Puffenberger *et al.*, 2012).

### Neuropathology

In Case 1, the fixed brain was first examined macroscopically, and then microscopically using multiple paraffin embedded blocks. In Case 2, a limited number of paraffin blocks were received from the NICHD Brain and Tissue Bank for Developmental Disorders at the University of Maryland. Standard histological and immunohistological methods were used as described previously (Harding *et al.*, 2015) with the addition of immunocytochemistry for choline acetyltransferase (Chat22 goat polyclonal antibody 1:100, #AB144P, Millipore).

### WDR73 expression in human brain: tissue processing and immunohistochemistry

Frontal neocortex and cerebellar specimens were obtained from three control subjects who died of non-neurological causes (mean age 5.8 years; two male, one female; Brain and Tissue Bank for Developmental Disorders, University of Maryland, mean post-mortem interval = 14 h, range 11–16 h). Cortical cytoarchitecture of specimens was intact. Human tissue specimens were either fixed in 4% paraformaldehyde and paraffin embedded or flash frozen at −70°C. Tissue blocks were microtome sectioned at 7 µm and five representative sections per case were probed with WDR73 antibodies (Novus Biologicals, rabbit polyclonal, 1:200) overnight at 4°C. Sections were then probed with biotinylated secondary antibodies for 1 h at room temperature and visualized using avidin-biotin conjugation (Vectastain ABC Elite; Vector Labs) with 3,3'-diaminobenzidine. Dehydrated sections were mounted with coverslips (Permount). Light-microscopy images were acquired using a Leica DM4000 B microscope. All human tissue was obtained using protocols approved by the Temple University Institutional Review Board.

### Renal pathology

Tissue was processed by standard methods for light microscopy, direct immunofluorescence, and electron microscopy. All materials were examined by a renal pathologist (A.F.), and an integrated diagnosis was rendered.

### Cell culture

Primary cultures of dermal fibroblasts from NCS subjects (homozygous c.888delT) and their heterozygous parents were established by the Coriell Institute (Camden, NJ), and normal human dermal fibroblasts (NHDFs) were purchased from Coriell. Fibroblasts and HEK-293T cells (ATCC) were cultured at low passage (≤10) in Dulbecco’s modified Eagle medium (DMEM) (Life Technologies) with 10% foetal bovine serum (FBS) at 37°C and 5% CO_2_. Mouse neural progenitor cells were derived from the subventricular zone of C57BL/6 postnatal Day 1 mice as described previously (Orlova *et al.*, 2010). Mouse neural progenitor cells were cultured on poly-D-lysine coated plates in DMEM/F12 with 1% FBS, 1% N-2 supplement, 1% penicillin/streptomycin, fibroblast growth factor, and heparin and maintained at 37°C. Human astrocytes were obtained from resected temporal lobe epilepsy specimens (courtesy D. Kolson M.D., Ph.D., University of Pennsylvania). For immunoblotting shown in Fig. 4 and immunofluorescence shown in Supplementary Fig. 2, NHDFs and astrocytes were grown in DMEM (Life Technologies #11962-084), 5% FBS, 1% penicillin/streptomycin, 5 ml per 500 ml total volume of AGS solution (from ScienCell, cat # 1852). U87 glioma lines were obtained (courtesy D. O’Rorke M.D., Ph.D., University of Pennsylvania) and maintained in DMEM (Life Technologies, #11965-084, 10% FBS, 1% penicillin/streptomycin). Human neural stem cells (H-9 derived, Life Technologies; cat #10142-01) were grown and maintained in StemPro NSC medium (KnockOut DMEM/F-12, GlutaMAX™ supplement, bFGF, EGF, and StemPro Neural Supplement, #A10508-0) at 37°C and 5% CO_2_.

### Cell proliferation assay

Dermal fibroblasts seeded at equal densities on 6- or 12-well plates in DMEM with 10% FBS were cultured (medium replaced every 2–3 days) until NHDFs reached confluency (Fig. 4) or until heterozygous parent cells reached 50–75% confluency (Supplementary Fig. 1). Cells were then washed with phosphate-buffered saline (PBS; pH 7.2), fixed for 15 min with 4% paraformaldehyde in PBS at 21°C, washed with sterile water, stained with 0.1% crystal violet in 10% ethanol for 20 min, and washed three times with sterile water. Plates were dried and scanned on an Epson flatbed scanner. Cells were then extracted with 10% acetic acid and the absorbance of the extract was measured with a spectrophotometer at 590 nm. Data were analysed using Student’s *t*-test (α = 0.05) with Microsoft Excel.

**Figure 1:**
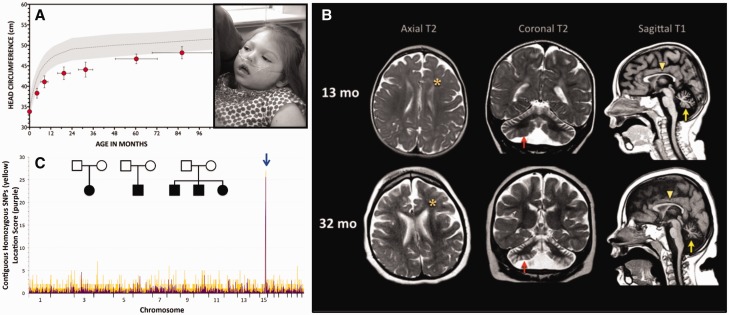
**Nephrocerebellar syndrome**. (**A**) Postnatal brain growth is slow in children with NCS (grey-shaded area = normal head circumference for age, mean ± 2 SD; red circles = NCS head circumference, mean ± SD). *Inset*: Children with NCS have no distinctive dysmorphic features, but the forehead characteristically recedes, reflecting relative hypoplasia of the cerebral cortex; post-mortem brain weights are only 50–60% of normal (photos used with parental permission). (**B**) MRI at 1.5 T (left to right: axial T_2_, coronal T_2_, sagittal T_1_) shows underdeveloped frontal lobes (asterisks), thin corpus callosum (yellow arrowheads), and severe atrophy of the cerebellar hemispheres (red arrows) and vermis (yellow arrows). Comparable images of the same NCS child, taken at 13 months (*upper panel*) and 32 months (*lower panel*), reveal progressive degeneration of cerebellar tissue (arrows). (**C**) Initial SNP genotyping of five children (*inset*) mapped recessive NCS to an 8.26 Mb autozygous block of DNA on chromosome 15 (blue arrow). After the initial mapping, we identified nine additional patients that allowed us to narrow the shared homozygous interval to 3.96 Mb. Yellow signal indicates the number of contiguous SNPs shared among NCS individuals. For each block of shared homozygous SNPs, purple signal shows the cumulative two-point LOD score (‘location score’), a relative probability, based on population allele frequencies, that the disease gene resides in the homozygous block. Subsequent genotyping of all 27 NCS individuals refined the shared locus to 700 kb, which contained the novel *WDR73* c.888delT mutation homozygous in all affected children.

### Immunofluorescence microscopy

Fibroblasts and HEK-293T cells cultured on uncoated German glass coverslips were rinsed with PBS at 37°C and fixed with 4% paraformaldehyde at 21°C for 15 min. Cells were then prepared for immunofluorescence microscopy and documented as described previously (Puffenberger *et al.*, 2012) using the following primary antibodies: anti-WDR73 (1:50) [Sigma HPA039357 rabbit polyclonal; same as Novus NBP1-82219; validated by short hairpin (sh)RNA knockdown of WDR73 and overexpression of recombinant WDR73 in HEK-293 T cells; Figs 6C–E and 7E]; anti-WDR73 (1:50) (Thermo PA5-25221 rabbit polyclonal; validated against overexpression of WDR73; Fig. 6C–E); anti-α-tubulin (1:1500) (Sigma T6074); anti-β-actin (1:200) (Sigma A1978); anti-V5 (1:500) (Life Technologies 377500). Secondary antibodies: Alexa Fluor® 488- (green) or 594- (red) conjugated goat anti-rabbit or mouse IgG (1:400) (Life Technologies). For Supplementary Fig. 2, cells were fixed in ice-cold 4% paraformaldehyde and probed with WDR73 antibodies (Novus NBP1-82219) overnight at 0°C and then with Texas Red® or FITC conjugated anti-rabbit IgG secondary antibody. Coverslips were mounted with Fluoromount-G™ (Electron Microscopy Sciences). To synchronize fibroblasts in mitosis, double-thymidine block was performed as described previously (Robitaille *et al.*, 2013). All experiments were replicated two to five times.

MitoTracker® Red was used to label the mitochondrial network. Briefly, cells were incubated with MitoTracker® Red CMXRos (300 nM) at 37°C/5% CO_2_ for 30 min, washed with PBS, and fixed in 4% paraformaldehyde in PBS for 15 min at 37°C. Cells were then permeabilized in acetone at −20°C for 5 min, washed with PBS, and counterstained with DAPI (4',6-diamidino-2-phenylindole; 1.5 µg/ml; Santa Cruz Biotechnology).

### Co-immunoprecipitation and immunoblotting

Frozen human tissues (cortex and cerebellum) and cell lines (Figs 4A, 7E, and Supplementary Fig. 4E) were lysed in RIPA buffer (50 mM Tris HCl pH 8.0; 150 mM NaCl; 1% NP-40; 0.5% sodium deoxycholate, 0.1% SDS, protease and phosphatase inhibitors). Protein (30 µg) was separated on 4–15% SDS-PAGE Tris-Glycine gels (Bio-Rad), transferred onto PVDF membranes and probed with anti-WDR 73 antibodies (Novus; 1:1000) overnight at 4°C and horseradish peroxidase-conjugated secondary antibodies (GE Healthcare) for 1 h at room temperature, and visualized with ECL or ECL Plus (GE Healthcare). Anti-GAPDH (Cell Signaling) labelling was used to ensure equal protein loading. Each set of experiments was repeated twice. For the remaining figures, cells were lysed in ice-cold RIPA or CelLytic M (Sigma) lysis buffer with protease and phosphatase inhibitor cocktail (Roche) and 1 mM phenylmethanesulfonylfluoride (PMSF; Sigma), separated by SDS-PAGE on 4–12% Bis-Tris gels (Life Technologies), stained with Coomassie Brilliant Blue (for mass spectrometry), or transferred to 0.45 µm nitrocellulose (Bio-Rad). Immunoblots were prepared and documented as previously described (Puffenberger *et al.*, 2012) with the following antibodies: anti-FLAG M2 (1:1000) (Sigma); anti-V5 (1:5000) (Life Technologies); anti-WDR73 (1:100) (Sigma HPA039357); anti-β-actin (1:250 000) (Sigma A1978); anti-α-tubulin (1:2000) (Sigma T6074); anti-γ-tubulin (1:1000) (Sigma), anti-COXIV, anti-CAD, anti-phosphoCAD, anti-HSP-70, anti-HSP-90, anti-p70 S6 kinase, anti-β-tubulin (1:1000) (Cell Signaling). Immunoblots were stripped and reprobed using Restore Plus (Thermo) per the manufacturer's instructions.

Co-immunoprecipitation assays were performed as described elsewhere (Puffenberger *et al.*, 2012) with the following exceptions: clarified lysates were incubated with the anti-FLAG M2 affinity gel for 2 h at 4°C followed by 3–4 15-min washes in wash buffer with 2.5–5% Tween-20 (Sigma), protease inhibitors, phosphatase inhibitors, and 1 mM PMSF. Immunoprecipitated proteins were eluted from the affinity gel with 3× FLAG peptide (150 ng/µl), and immunoblotted as described above.

### Complementary DNA constructs and transfection

Human *WDR73* (NM_032856.2) and *CAD* (NM_004341.3) cDNAs amplified from HEK-293T total RNA using RT-PCR (SuperScript® III reverse transcriptase, Life Technologies; Phusion DNA polymerase, NEB) were cloned into pENTR/D-TOPO (Life Technologies) and mutations indicated in the ‘Results’ section were introduced by site-directed mutagenesis (QuikChange® II, Agilent). Constructs were then recombined into pCAG/FLAG/RFC-A (Puffenberger *et al.*, 2012) using LR Clonase II (Life Technologies) for expression of N-terminal FLAG-tagged fusion proteins. To generate plasmids for WDR73 and CAD C-terminal V5 fusion proteins, cDNAs were cloned in-frame directly into pcDNA3.1/TOPO/V5-DEST or recombined into pcDNA3.2/V5-DEST (Life Technologies). All constructs were verified by Sanger sequencing.

HEK-293T cells were transfected with FuGENE6 (Promega) according to the manufacturer’s protocol and cultured for 40–48 h before immunofluorescence microscopy, immunoblotting, or coimmunoprecipitation. NCS patient fibroblasts were transfected by nucleofection using the Amaxa® Human Dermal Fibroblast Nucleofector® Kit (Lonza) and a Nucleofector 2b NHDF program optimized for cell viability following nucleofection.

## Results

### Genetic mapping

Using 10 000- and 50 000-marker SNP microarrays, we genotyped five affected Amish children and identified a shared 8.26 Mb autozygous block on chromosome 15 that was further refined with nine additional NCS patients to a 3.96 Mb autozygous block (flanked by rs2048271 and rs959181) that contained 69 genes (Fig. 1C). A single child from Indiana had a rare recombination event between rs4359399 and rs713468 that narrowed the minimal autozygous region to 700 kb. Exome sequencing of DNA from seven children with NCS identified a novel homozygous single base pair deletion (*WDR73* c.888delT) in all affected individuals. We used Sanger sequencing to confirm that the *WDR73* c.888delT frameshift was homozygous in all 27 NCS children and heterozygous in parents.

An additional 800 children with idiopathic nephrotic syndrome were screened using microfluidic multiplex PCR (Fluidigm) and next-generation sequencing (Halbritter *et al.*, 2012; Sadowski *et al.*, 2015). A second homozygous frameshift variant, *WDR73* c.766dupC (p.Arg256Profs*18) was identified in a Bulgarian child with NCS. The *WDR73* c.766dupC variant was also identified recently in an unrelated child of Turkish descent with NCS (Colin *et al.*, 2014).

The 3.96 Mb Amish autozygous block contained a second truncating variant in *WHAMM* (c.1264_1270delATAAAAG) (NM_001080435.2) on the same haplotype as the *WDR73* mutation. Genotype analysis of all affected individuals revealed that the Indiana Amish NCS patient described above (3 years old) was homozygous for only the *WDR73* variant, and heterozygous for the *WHAMM* mutation. Her clinical course was consistent with NCS (Fig. 1A) although she had not yet developed nephrotic syndrome. As kidney disease onset is variable in NCS/Galloway-Mowat syndrome, this finding was not unusual. Three additional affected siblings, not available for follow-up, were also considered likely to be homozygous for only the *WDR73* mutation and wild-type for the *WHAMM* mutation (genotypes inferred from parents and an unaffected sister). We were unable to identify any clinical differences between these three individuals and those who were doubly homozygous for both *WDR73* and *WHAMM* variants. In our single non-Amish proband from Bulgaria, we excluded *WHAMM* mutations through Sanger sequencing.

### Phenotype of nephrocerebellar syndrome on the Galloway-Mowat spectrum

Table 1 summarizes core phenotypic features of 30 NCS patients (27 confirmed and three inferred *WDR73* c.888delT homozygotes) ages 1.4 to 28 years (mean age 9.2 years). Irritability, congenital roving nystagmus, and visual impairment were early postnatal signs of NCS. Head growth was slow (Fig. 1A and B) and psychomotor development was severely delayed in all domains. Affected children did not effectively communicate or use their hands, and only 10% achieved independent ambulation (Table 1). Most children with NCS developed extrapyramidal movements that included a combination of axial dystonia and limb chorea. Severe visual impairment was associated with progressive optic atrophy. The majority of affected children had irregular or reversed sleep-wake patterns.

**Table 1:** Clinical features of 30 individuals with NCS

Mean age in years (range)	9.2 (1.4–28.0)
**Neurological phenotype (% affected)**	
Roving eye movements	100%
Visual impairment/optic atrophy	93%
Non-communicative	97%
No independent ambulation	90%
No independent sitting	90%
Central hypotonia	87%
Extrapyramidal movement disorder	87%
Microcephaly^a^	80%
No purposeful hand use	77%
Epilepsy	41%
**Renal phenotype (% affected)**	
Nephrotic syndrome^b^	57%
Renal insufficiency/failure^c^	50%
Untimely death^e^	47%
Mean age of death in years (range)	11.0 (2.7–28.0)

Table contains data from 27 confirmed patients and three inferred homozygous *WDR73* c.888delT genotype.

^a^Head circumference ≤2 SD below normal for age.

^b^Seventeen subjects had clinical signs of nephrosis (oedema, ascites) but only 11 had confirmatory laboratory testing (i.e. serum albumin, urine protein/creatinine ratio).

^c^Fifteen subjects had clinical signs of renal failure (oedema, anemia, oliguria/anuria, etc.) but only nine had confirmatory laboratory testing (i.e. urea nitrogen, creatinine, etc.).

^e^In most cases due to complications of renal failure.

Multifocal seizures began during infancy or early childhood in 41% of affected children. EEGs were characterized by slow, disorganized background, absent posterior (occipital) rhythm, poor sleep-wake differentiation, multifocal sharp and spike-slow wave discharges from various cortical regions and rarely, high-voltage modified hypsarrhythmia. Serial MRIs revealed diffuse cerebral atrophy, thinning of the corpus callosum, and hypoplasia and progressive atrophy of cerebellar tissue (Fig. 1B).

More than half of NCS individuals (57%) developed steroid non-responsive, fluctuating proteinuria (mean urine protein:creatinine ratio 12.0 mg:mg, range 3.5–20.0 mg:mg; normal reference range 0.0–0.2 mg:mg) during early childhood (Fig. 3A) in association with pitting oedema, hypoalbuminaemia, renal insufficiency, non-regenerative anaemia, or some combination of these (Table 1). Among 30 NCS subjects, 14 (47%) died between ages 2.7 and 28 years (mean 11.0 ± 8.6 years), in most cases from complications of renal failure.

### Neuropathology

**Figure 2:**
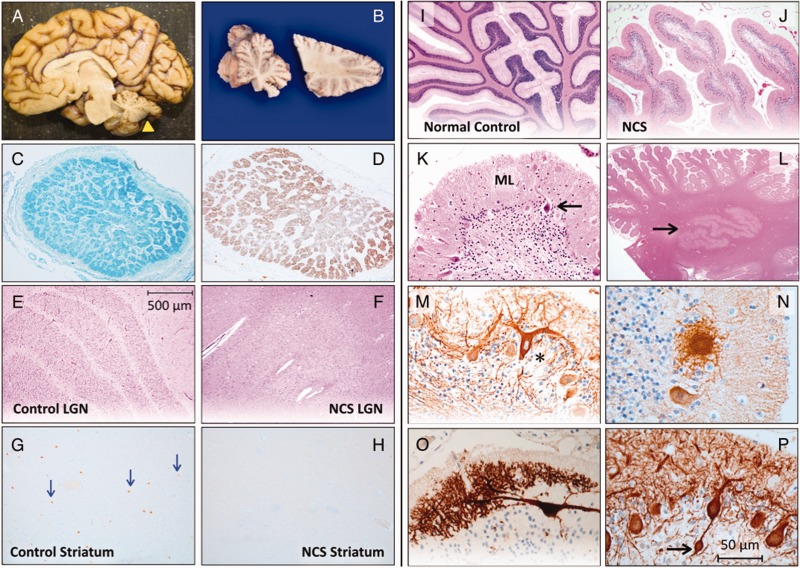
**Neuropathology of NCS**. (**A**) A child with NCS died from complications of renal failure at 3.5 years of age. Total brain weight was 636 g (60% of expected) and the hindbrain weighed 7.9% of the total (expected 12%). (**B**) The cerebellum (yellow arrowhead in **A**) was small, firm and sclerotic. Luxol Fast Blue (**C**) and GFAP (**D**) stains show atrophy and gliosis within cross-sections of the optic nerve. (**E**) The normal hexalaminar structure of the lateral geniculate nucleus (LGN) is compared with the lateral geniculate nucleus of an NCS child (**F**), that is nearly devoid of magnocellular neurons, parvocellular neurons, and laminae. (**G**) Choline acetyltransferase staining of normal striatum reveals several large cholinergic interneurons (arrows), which are absent from a comparable histological section of NCS striatum (**H**). (**I**) Normal cerebellar cortex stained with haematoxylin-eosin is compared to that of NCS cerebellum (**J**), which has short, stubby folia with sparse nuclei in the granule cell layer. (**K**) Higher magnification shows severe depletion of granule cells with relative preservation of Purkinje neurons (arrow) and a thin, hypercellular molecular layer (ML). (**L**) Dentate nuclei (arrow) have normal structure and cellularity. (**M**) Deafferented Purkinje neurons (asterisk) stained for calbindin and neurofilament have ‘weeping’ dendrite configurations, profusion of dendritic elements into ‘asteroid bodies’ (**N**) and other complex branching patterns (**O**)**,** and (**P**) bulbous (‘torpedo’) swelling within proximal axons (*arrow*).

Brain tissue was examined post-mortem from two affected females who died from complications of renal failure at ages 3.5 (Case 1) and 9.3 years (Case 2); pathological findings were similar. Both brains were small, with fixed weights of 636 g (normal for age, 1100 g) and 681 g (normal for age, 1250 g), respectively, and most striking were small sclerotic cerebella with prominent folia (Fig. 2A and B). The hindbrain of Case 1 weighed 50 g, only 7.9% of total brain weight (expected 12%); this was accompanied by ventricular dilatation, a thin corpus callosum, and atrophic optic nerves (Fig. 2C and D).

The cerebral cortex showed normal lamination with no obvious gliosis or cell death. Pathological changes were found in the visual system, striatum, and cerebellum. Optic nerve atrophy was confirmed microscopically (Fig. 2C and D), and both magnocellular and parvocellular neurons of lateral geniculate nuclei were nearly absent, obliterating the normal six-layer organization (Fig. 2E and F). No degenerative changes or gliosis were present in the basal ganglia, but the magnocellular cholinergic interneurons in the striatum were small and scarce (Fig. 2G and H). By comparison, medium spiny neurons appeared normal.

The cerebellum in both affected children was markedly abnormal. Folia of the cerebellar hemispheres and vermis were short and stubby (Fig. 2I and J) and granule cells were profoundly depleted (Fig. 2K). Purkinje neuron morphology was relatively spared but these cells were decreased in number and crowded together. There was excessive Bergmann gliosis. The overlying molecular layer was thin and hypercellular, comprised of Bergmann glia, misplaced NeuN-positive granule cells, and rare Purkinje neurons (Fig. 2K). Calbindin and neurofilament immunostaining of Purkinje neurons revealed spherical dendritic swellings covered in fine spikes (‘asteroid bodies’), dysmorphic dendritic trees forming ‘weeping willow’ arrangements, and occasional Purkinje cell bodies covered in somal sprouts suggestive of deafferentation (Fig. 2M–O). Globose (‘torpedo’) swellings of proximal Purkinje cell axons suggested ongoing cell degeneration (Fig. 2P). Alpha-tubulin staining revealed occasional binucleate Purkinje neurons (Supplementary Fig. 1E and F). Neurofilament staining demonstrated preservation of basket cells. Dentate nuclei were unaffected (Fig. 2L).

### Renal pathology

**Figure 3:**
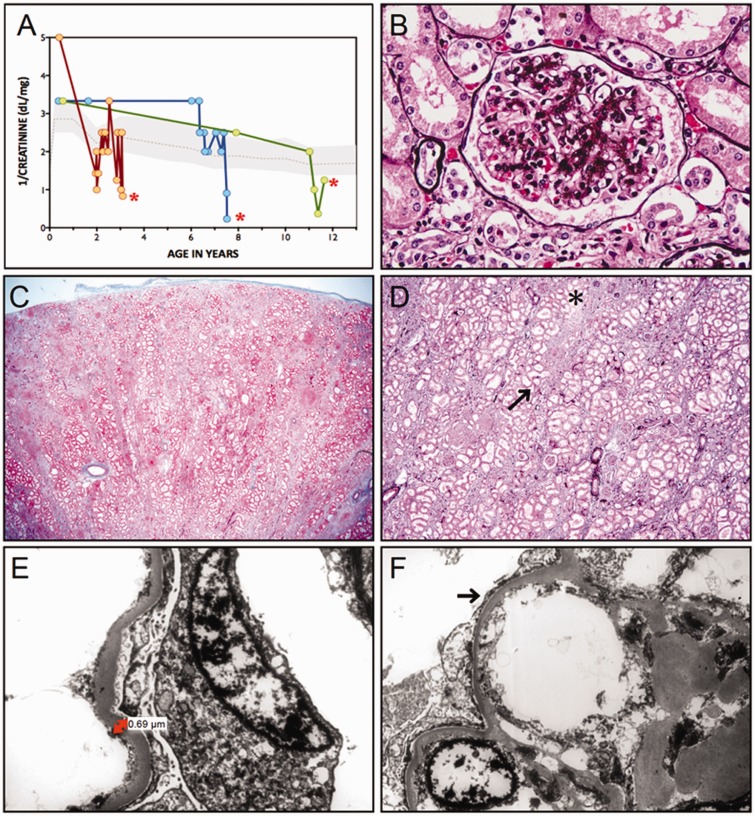
**Kidney disease.** (**A**) Variable onset of nephrosis is followed by progressive loss of glomerular function, shown as 1/serum creatinine (dl/mg), in three with NCS who died (red asterisks) from complications of renal failure at ages 3.5, 8, and 12 years. (**B**) Jones’ stain reveals both global and segmental sclerosis of glomeruli. (**C**) ‘Striped’ fibrosis is seen throughout the kidney interstitium. (**D**) At higher magnification, linear tracks of fibrosis (arrow) correspond to adjacent tubular atrophy (asterisk). (**E**) Electron micrographs show a 3- to 4-fold thickening of the glomerular basement membrane (normal for age, 0.15 µm) accompanied by (**F**) effacement and microvillus transformation of podocyte foot processes (*arrow*).

Renal biopsies in three affected children showed similar morphological features consistent with primary focal segmental glomerulosclerosis (Fig. 3). Nearly 60% of glomeruli exhibited severe sclerosis; remaining glomeruli showed segmental sclerosis, hyalinosis and adhesion to Bowman’s capsule. A ‘tram-track’ configuration was occasionally seen in the glomerular basement membrane (Jones silver stain) (Fig. 3B). Striped fibrosis with proportional tubular atrophy affected 40–50% of the renal interstitium (Fig. 3C and D). The glomerular basement membrane (mean 437 nm, range 345–686 nm) was 3- to 4-fold thicker than normal (average glomerular basement membrane thickness for age 150 nm) (Fig. 3E). Podocyte foot processes were 80–90% effaced in association with extensive microvillus transformation (Fig. 3F).

### Expression of *WDR73* variants in cells and tissues

The *WDR73* c.888delT variant is predicted to result in substitution of Phe296 with leucine, followed by 24 frame-shifted amino acids and truncation of full-length WDR73 from 378 (42 kDa) to 320 (35.1 kDa) amino acids. *WDR73* c.766dupC is predicted to result in a proline substitution at Arg256, followed by 16 frame-shifted amino acids and premature termination after amino acid 272, producing a 29.8 kDa protein (Colin *et al.*, 2014). Structural homology modelling of full-length WDR73 using WDSPdb/ds (Wang *et al.*, 2015) predicts six beta-propellar WD repeat domains (Colin *et al.*, 2014). Phe296, changed to leucine in WDR73 p.Phe296Leufs*26 and deleted in p.Arg256Profs*18, is a protein–protein interaction hotspot (WDSPdb/ds; Wang *et al.*, 2015) on the surface of the fifth WD repeat. This suggests that protein–protein interactions are altered by both pathogenic WDR73 variants.

**Figure 4:**
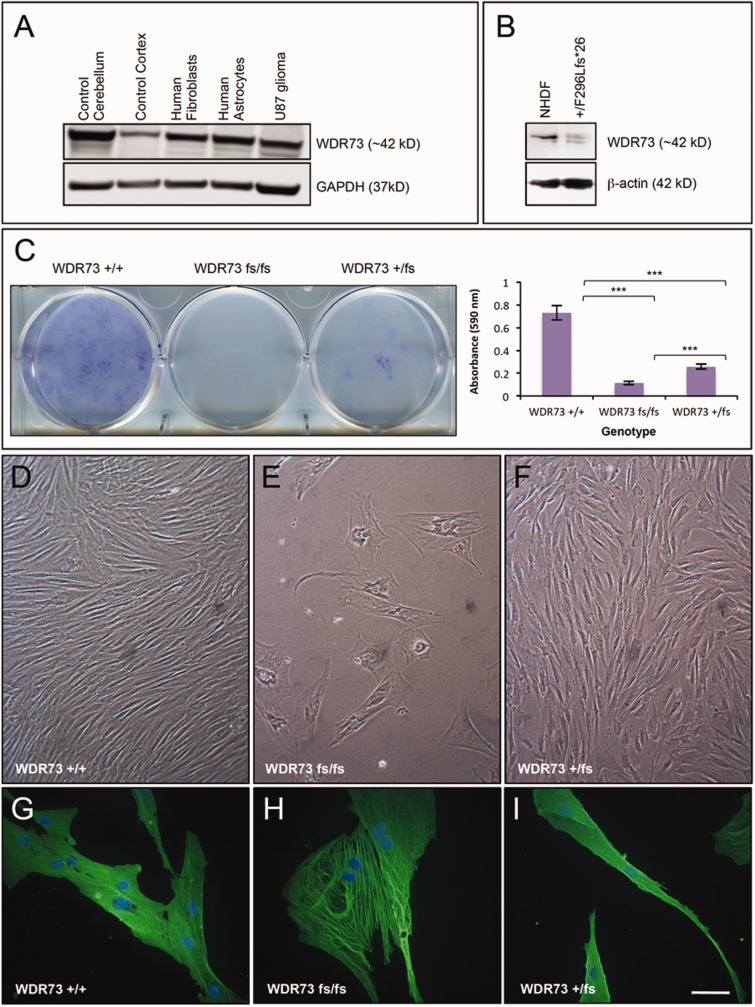
**WDR73 expression in human brain and NCS cells.** (**A**) Immunoblot of WDR73 expression in: lanes 1 and 2, normal post-mortem human cerebellum, cerebral cortex; lanes 3–5, cultured human cells as indicated. (**B**) Immunoblot of WDR73 from NHDFs and dermal fibroblasts from a heterozygous (WDR73 +/p.Phe296Leufs*26) parent of a child with NCS. Note the additional lower molecular mass band for the truncated WDR73 in the heterozygous parent’s cell lysate. (**C**) NCS (p.Phe296Leufs*26) fibroblasts (fs/fs) grow poorly in primary culture. *Left*: Crystal violet cell proliferation assay demonstrating reduced proliferation of dermal fibroblasts from a child with NCS (WDR73 fs/fs) relative to the heterozygous parent’s cells (WDR73 +/fs) and NHDF (WDR73 +/+) plated at the same density. *Right*: Quantification of cell proliferation by absorbance of extracted crystal violet at 590 nm (*n = *4) (***P < 0.001). (**D–F**) Phase contrast comparison of growth characteristics and morphologies of NHDF (WDR73 +/+), NCS (p.Phe296Leufs*26; WDR73 fs/fs), and heterozygous parent fibroblasts (p.Phe296Leufs*26; WDR73 +/fs) seeded at equal densities and grown under the same conditions on the same 6-well plate. (**G–I**) Anti-β-actin immunofluorescence microscopy of NHDF (WDR73 +/+), NCS (p.Phe296Leufs*26; WDR73 fs/fs), and heterozygous parent fibroblasts (p.Phe296Leufs*26; WDR73 +/fs). Nuclei were counterstained with DAPI. Scale bars: **D–F** = 140 µm; **G–I** = 55 µm.

In post-mortem brain tissue, wild-type WDR73 immunoreactivity was observed within pyramidal cells of the cerebral cortex and hippocampus (Supplementary Fig. 1A and B), and within Purkinje and granule cell neurons of the cerebellum (Supplementary Fig. 1C), where it largely localized to cytoplasm within somatodendritic domains. Western blot analysis revealed full-length WDR73 (42 kDa) in human cerebral cortex and cerebellum as well as fibroblasts, astrocytes, and U87 glioma cells (Fig. 4A; see also Supplementary Fig. 2).

NHDFs express full-length WDR73 (Fig. 4A and B; see also Colin *et al.,* 2014) whereas fibroblasts cultured from WDR73 p.Phe296Leufs*26 heterozygotes display two WDR73 immunoreactive bands (∼42 kDa and 35 kDa), each of reduced abundance relative to the single band observed in NHDFs (Fig. 4B). We were unable to culture enough NCS proband (p.Phe296Leufs*26) fibroblasts to reliably detect a WDR73 band by immunoblotting, but would predict reduced abundance of both WDR73 p.Phe296Leufs*26 and p.Arg256Profs*18 based on instability of the recombinant versions of these proteins (Fig. 6F and G).

### Cell proliferation assay

Fibroblasts from NCS patients grow poorly in primary culture, especially if cultured at low density and beyond 2–3 passages. Over a 2-week period, fibroblasts from an NCS proband (p.Phe296Leufs*26) and her heterozygous father grew to 16% and 35% of NHDF density, respectively (*n = *4 replicates) (Fig. 4C). If seeded more densely (5000 cells per well in a 12-well plate) and cultured until NHDFs reached full confluency, NCS proband and heterozygous parent cells grew to 43.5% and 78% of NHDF density, respectively (*n = *4) (Supplementary Fig. 1G).

### Abnormal cell cycle in NCS fibroblasts

**Figure 5:**
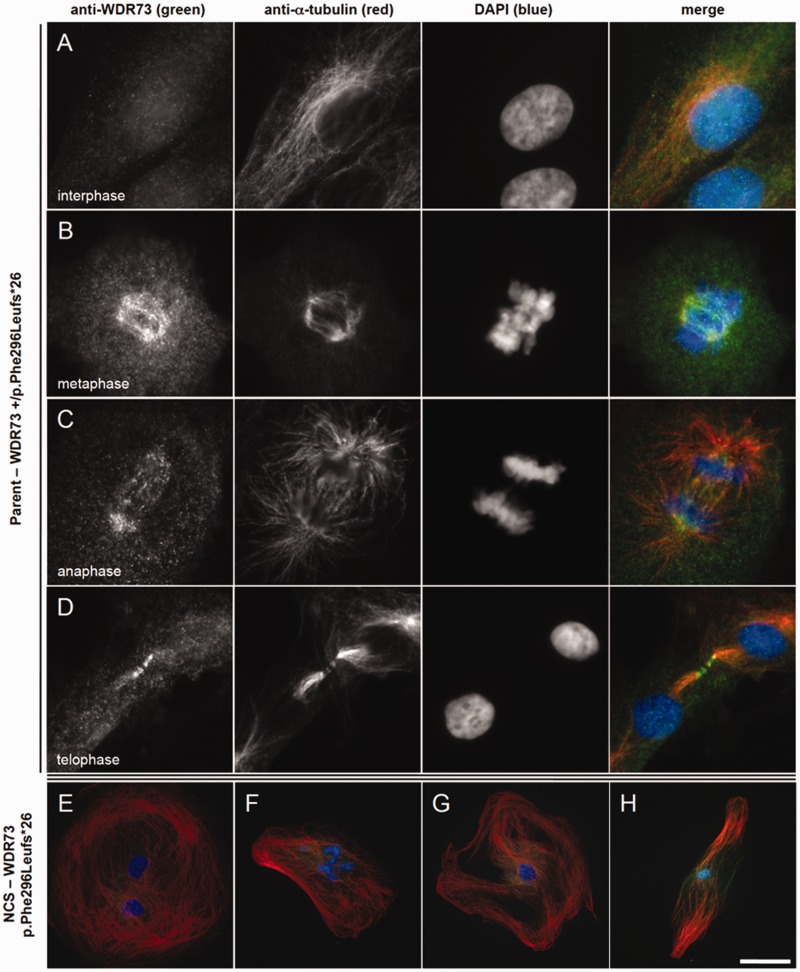
**WDR73 immunoreactivity in fibroblasts from a child with NCS and a heterozygous parent.** (**A–D**) Heterozygous (WDR73 +/p.Phe296Leufs*26) parent fibroblasts. (**A**) Anti-WDR73 (green) immunoreactivity is diffuse and cytosolic during interphase (anti-α-tubulin, red). (**B**) WDR73 immunoreactivity localizes to the spindle poles and spindle microtubules during metaphase. (**C**) WDR73 immunoreactivity at the mitotic spindle poles, kinetochore microtubules and central spindle microtubules during anaphase. (**D**) WDR73 immunoreactivity localizes to the midbody microtubules apposing the Flemming body (stem body) during telophase. (**E–H**) WDR73 immunoreactivity is weak and cytosolic in NCS fibroblasts homozygous for WDR73 p.Phe296Leufs*26. (**E** and **F**) Twenty-two per cent of NCS fibroblasts observed were binucleate and an additional 20% displayed abnormal nuclear morphology (bi-/multi-lobed nuclei, budding micronuclei, or nucleoplasmic bridges). (**G** and **H**) Additional NCS fibroblasts displaying the range of microtubule network morphology observed. Note that the magnifications are reduced in **E–H**. Scale bars: **A–D** = 10 µm; **E–F** = 50 µm; **G–H** = 90 µm.

Fibroblasts from NCS probands (p.Phe296Leufs*26) were substantially larger (up to 280 µm in diameter) than NHDFs and heterozygous parent fibroblasts (Figs 4D–I and 5E–H). Using immunofluorescence microscopy, we always observed NCS proband fibroblasts in interphase; they did not appear to progress through other phases of the cell cycle. Cytoplasmic WDR73 immunoreactivity was weak in these cells and difficult to distinguish from background (Fig. 5E–H).

Using live-cell phase contrast microscopy, we occasionally identified large, distorted structures resembling mitotic profiles (data not shown), but none were observed in normal telophase/cytokinesis. Instead, 24% of proband cells (*n = *125) observed were bi- or multi-nucleate (Figs. 4H and 5E) and another 20% displayed abnormal nuclear morphology (bi- or multi-lobed nuclei, budding micronuclei, and nucleoplasmic bridges) (Fig. 5F). In contrast, only 19.2% and 5.5% of nuclei were abnormal in heterozygous parent fibroblasts (*n = *104) and NHDFs (*n = *309), respectively. Actin and mitochondrial networks appeared normal in NCS proband fibroblasts (Fig. 4G–I and Supplementary Fig. 1H–J, respectively), although stress fibres were abundant (Fig. 4H).

### WDR73 associates with the mitotic microtubules during cell division

Reduced proliferation and early senescence of NCS proband fibroblasts suggests that WDR73 plays a fundamental role in the cell cycle. Accordingly, we determined the subcellular localization of WDR73 immunoreactivity across the cell cycle in NHDFs (Supplementary Fig. 3; see also Colin *et al.*, 2014) and parent fibroblasts heterozygous for WDR73 p.Phe296Leufs*26 (Fig. 5). There were no substantial differences in WDR73 immunoreactivity between NHDF and heterozygous parent fibroblasts. During interphase, WDR73 immunoreactivity was diffusely cytoplasmic (Fig. 5A). As cells transitioned into metaphase, WDR73 immunoreactivity associated strongly with α-tubulin at the spindle poles and spindle microtubules (Fig. 5B). During anaphase, WDR73 localized to spindle poles, kinetochore microtubules, and interpolar microtubules (Fig. 5C). During telophase, WDR73 immunoreactivity transitioned from the spindle poles along the interpolar microtubules to the central spindle microtubules, to concentrate at the midbody microtubules apposing the stem body, where central spindle microtubules arising from opposite poles of the cell overlap (Fig. 5D). These results were consistent across all cells observed in mitosis. As described above, fibroblasts from patients homozygous for WDR73 p.Phe296Leufs*26 could not be observed in any phase of cell cycle outside of interphase (Fig. 5E–H).

**Figure 6:**
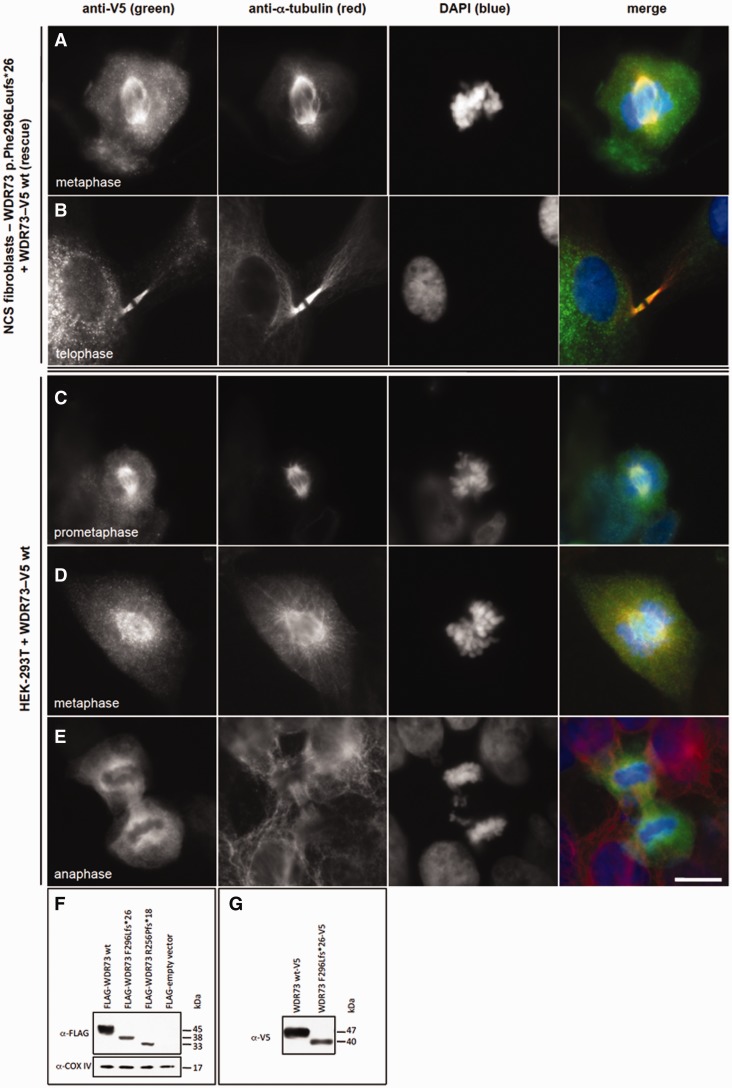
**Recombinant WDR73-V5 fusion protein rescues cell cycle defect in NCS patient fibroblasts.** (**A** and **B**) Anti-V5 immunofluorescence (green) demonstrates that WDR73 C-terminal V5 fusion protein (WDR73–V5) overexpressed in NCS patient fibroblasts localizes to the mitotic microtubules (anti-α-tubulin) during metaphase (**A**) and telophase (**B**), rescuing the cell cycle defect in these cells. (**C–E**) Overexpression of WDR73 C-terminal V5 fusion protein (WDR73-V5) in HEK-293T cells. Anti-V5 immunofluorescence is in green; anti-α-tubulin in red. During pro-metaphase (**C**) and metaphase (**D**) recombinant WDR73-V5 colocalizes with α-tubulin at the mitotic spindle and aster microtubules. (**E**) WDR73-V5 localizes to the spindle poles, the kinetochore microtubules and the midzone microtubules during anaphase. Scale bars: **A–E** = 10 µm. (**F–G**) Western blots of recombinant N-terminal FLAG WDR73 fusion proteins (**F**) and WDR73 C-terminal V5 fusion proteins (**G**) overexpressed for 40–48 h in HEK-293T cells. Anti-COX IV was labelled as a protein loading control. The abundance of FLAG-WDR73 p.Phe296Leufs*26 (F296Lfs*26) was 2.6- to 5.6-fold lower and FLAG-WDR73 p.Arg256Profs*18 (R256Pfs*18) was 1.6- to 6.5-fold lower than that of FLAG-WDR73 wild-type (wt) across four replicates despite transfection of equivalent amounts of plasmid DNA, suggesting instability of the truncated proteins.

Transient overexpression of WDR73 wild-type C-terminal V5 fusion protein in fibroblasts from a patient homozygous for WDR73 p.Phe296Leufs*26 rescued the cell cycle defect. Transfected cells that presumably had not yet become binucleate were observed in metaphase (Fig. 6A) and telophase (Fig. 6B) within 5 days of transfection. Mitotic microtubules were only observed in transfected NCS patient fibroblasts. To validate our findings, we also overexpressed WDR73 wild-type C-terminal V5 fusion protein in HEK-293T cells. Recombinant WDR73-V5 localized robustly to the cytoplasm during interphase (data not shown), transitioned to the developing spindle poles and astral and spindle microtubules during prometaphase and metaphase (Fig. 6C and D), and was prominent at spindle poles, interpolar microtubules, and central spindle/midzone microtubules during anaphase (Fig. 6E).

### WDR73 protein–protein interactions

To explore the WDR73 interactome, we immunoprecipitated N-terminal FLAG-WDR73 wild-type (WT) and p.Phe296Leufs*26 fusion proteins overexpressed in HEK-293T cells. We then used Coomassie staining of eluted proteins, separated by SDS-PAGE, to identify co-immunoprecipitated proteins unique to FLAG-WDR73 expressing lysates as compared to those expressing only FLAG-peptide (‘empty vector’) (Supplementary Fig. 4A). Five unique bands present in FLAG-WDR73 expressing cells (*n = *4 replicates) were excised, digested with trypsin, and subjected to peptide mass fingerprinting with mass spectrometry (MALDI-TOF MS; Pennsylvania State University College of Medicine, Hershey, PA) (Supplementary Fig. 4A). Four of the five bands yielded high confidence protein IDs: α-tubulin 1B (TUBA1B), β-tubulin 4B (TUBB4B) (shared band), heat shock 70-kDa protein 1A/1B (HSP-70, encoded by *HSPA1A* and *HSPA1B*, respectively), heat shock protein HSP90 alpha isoform 2 (HSP-90), and CAD, the mTORC1-regulated multi-enzyme complex consisting of carbamoyl-phosphate synthetase 2, aspartate transcarbamylase, and dihydroorotase (Robitaille *et al.*, 2013).

**Figure 7:**
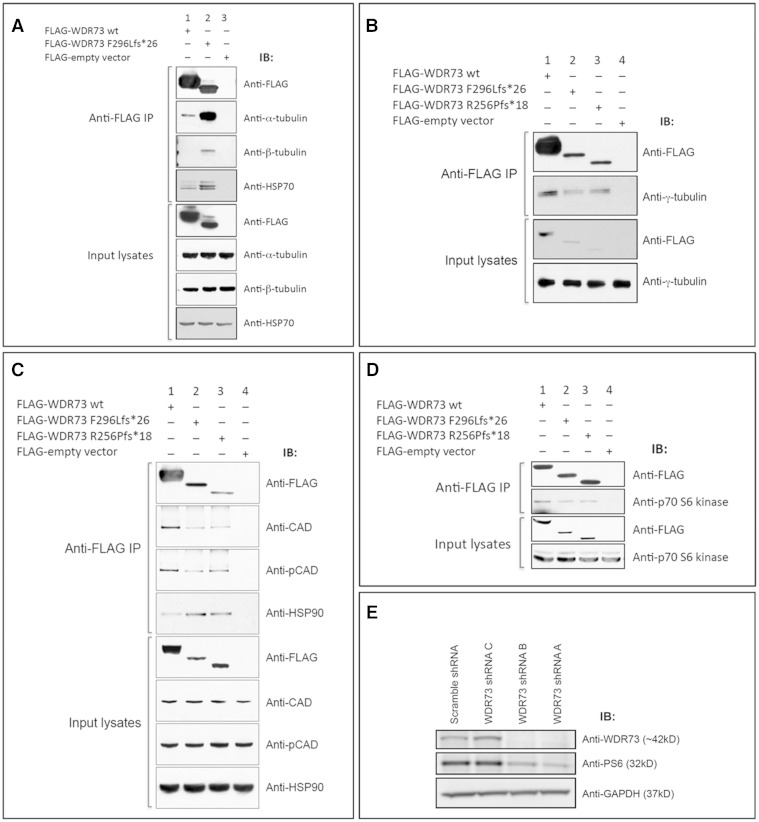
**WDR73 interacts with α-, β-, and γ-tubulin, CAD, HSP-70, HSP-90, and p70 S6 kinase.** (**A**) FLAG-WDR73 F296Lfs*26 (38 kDa) overexpressed in HEK-293T cells co-immunoprecipitated (IP) substantially more endogenous α- and β-tubulin and HSP-70 than FLAG-WDR73 wild-type (45 kDa). (**B**) FLAG-WDR73 wild-type, F296Lfs*26, and R256Pfs*18 each co-immunoprecipitated γ-tubulin at an abundance roughly proportional to abundance of the respective WDR73 construct. (**C** and **D**) Co-immunoprecipitation of (**C**) endogenous CAD, phospho-Ser^1859^ CAD, HSP-90, and (**D**) p70 S6 kinase from lysates of HEK-293T cells overexpressing the WDR73 constructs indicated. (**E**) Lentiviral transduction of shRNA plasmid constructs A and B targeting WDR73 in HEK-293FT cells successfully knocked down WDR73 and produced a concomitant decrease in ribosomal protein S6 phosphorylation. GAPDH was labelled as a protein loading control. This immunoblot was labelled with the same anti-WDR73 antibody (Novus) used for immunoblotting throughout the paper (validating the specificity of the antibody).

Interactions between WDR73 and α- and β-tubulin were confirmed by additional co-immunoprecipitation assays, which showed that FLAG-WDR73 wild-type co-immunoprecipitated both subunits from HEK-293T lysates (Fig. 7A and Supplementary Fig. 4B). Surprisingly, FLAG-WDR73 p.Phe296Leufs*26 (*n = *20) and p.Arg256Profs*18 (*n = *10) consistently co-immunoprecipitated substantially greater abundances of α- and β-tubulin, despite the reduced abundance of the truncated proteins (Fig. 7A and Supplementary Fig. 4B). By comparison, WDR73 immunoprecipitated γ-tubulin in relative proportion to the FLAG-WDR73 ‘bait’ abundance (*n = *6–9 replicates) (Fig. 7B).

WDR73 interaction with CAD was verified by FLAG-WDR73 co-immunoprecipitation of recombinant C-terminal V5 CAD fusion protein co-overexpressed in HEK-293T cells (*n = *4) (Supplementary Fig. 4C), and by co-immunoprecipitation of endogenous CAD from HEK-293T cell lysates overexpressing FLAG-WDR73 fusion proteins (*n = *7 for wild-type, p.Phe296Leufs*26; *n = *6 for p.Arg256Profs*18) (Fig. 7C). Co-immunoprecipitation of CAD was not affected by WDR73 protein truncation (Fig. 7C), suggesting an N-terminal CAD binding site on WDR73 (verified by co-immunoprecipitation with WDR73 and CAD deletion constructs; data not shown).

The WDR73:CAD interaction was not dependent upon phosphorylation of CAD (*n = *6) (Fig. 7C), and not diminished by changing CAD Ser1859 to alanine, which eliminates its site of phosphorylation by p70 S6 kinase via the mTORC1 pathway (Robitaille *et al.*, 2013) (*n = *2) (Supplementary Fig. 4D). We further determined that WDR73 interacts with p70 S6 kinase through coimmunoprecipitation of endogenous p70 S6 kinase by FLAG-WDR73 fusion proteins (*n = *11 for wild-type, p.Phe296Leufs*26; *n = *7 for p.Arg256Profs*18) (Fig. 7D). To probe the influence of WDR73 on mTORC1 signalling, we knocked down endogenous WDR73 in HEK-293FT and mouse neural progenitor cells and determined that phosphorylation of ribosomal protein S6 (a p70 S6 kinase substrate) was reduced without a decrease in S6 protein abundance (Fig. 7E and Supplementary Fig. 4E).

Relative to FLAG-WDR73 wild-type (*n = *7), truncated NCS WDR73 FLAG fusion proteins consistently co-immunoprecipitated substantially more endogenous HSP-70 (*n = *5) (Fig. 7A) and HSP-90 (*n = *7) from HEK-293T lysates (Fig. 7C), indicating that truncated proteins misfold and/or require additional chaperoning (Taipale *et al.*, 2010; Makhnevych and Houry, 2012).

## Discussion

We describe a novel truncating frameshift mutation in *WDR73* associated with a severe nephrocerebellar syndrome (NCS). NCS fits within the broad and evolving phenotypic spectrum of Galloway-Mowat syndrome (OMIM 251300), which is likely genetically heterogenous in aetiology (Pezzella *et al.*, 2010; Keith *et al.*, 2011; Ekstrand *et al.*, 2012; Colin *et al.*, 2014). Our neuropathological findings of delamination of the lateral geniculate nucleus, selective loss of cholinergic interneurons in the striatum and the absence of polymicrogyria and heterotopia in NCS patients are novel, and extend the pathological spectrum of Galloway-Mowat syndrome.

We provide the first description of the WDR73 interactome, as well as evidence, corroborating a recent report by Colin *et al.* (2014), that WDR73 interacts with mitotic microtubules and is critical for normal cell cycle progression, proliferation and survival of human fibroblasts, podocytes and neuronal subtypes. The two mutations associated with NCS we describe delete the sixth WDR73 C-terminal WD repeat domain and significantly alter the fifth, leading to loss of function, protein instability, and enhanced interactions with α- and β-tubulin. In fibroblasts, homozygous loss of WDR73 function results in decreased cell proliferation and abnormal cell cycle progression that can be rescued with wild-type WDR73 (Fig. 6). Whether the increase in apoptosis observed by Colin *et al.* (2014) in patient fibroblasts homozygous for WDR73 p.Tyr43* is a direct effect of WDR73 loss of function or a downstream effect of aborted cell cycle in bi- and multi-nucleated cells remains to be determined. Indeed, occasional binucleate cells were observed in post-mortem NCS cerebella (Supplementary Fig. 1).

Localization of wild-type WDR73 to the mitotic spindle, where it interacts with tubulin subunits, raises important questions about how WDR73 influences the function of the spindle and centrosome. Most genes linked to decreased brain size encode centrosomal proteins (e.g. *WDR62*) (Nicholas *et al.*, 2010; Yu *et al.*, 2010; Chen *et al.*, 2014) and dysfunction of the mitotic spindle is a well-established cause of microcephaly (Gilmore and Walsh, 2013). Abnormal nuclear morphologies observed in NCS proband fibroblasts are consistent with cytokinesis failure, indicating failure of the mitotic spindle (Normand and King, 2010). Overstabilization of interactions between pathogenic WDR73 fragments and tubulins may hinder normal movement of WDR73 along mitotic microtubules, slow spindle microtuble dynamics (Colin *et al.*, 2014), and/or slow the large amount of protein and vesicular trafficking along mitotic microtubules during cell cycle (Hu *et al.*, 2012; Agromayor and Martin-Serrano, 2013; Green *et al.*, 2013).

Slow proliferation of NCS fibroblasts might also reflect dysfunction of the mTORC1 signalling pathway. Our data suggest that WDR73 interacts in a complex with the mTORC1-regulated p70 S6 kinase and its natural substrate, CAD (Ben-Sahra *et al.*, 2013; Robitaille *et al.*, 2013). CAD is responsible for *de novo* pyrimidine synthesis during S-phase (Ben-Sahra *et al.*, 2013; Robitaille *et al.*, 2013). Knockdown of *Wdr73* in mouse neural progenitor cells resulted in decreased phosphorylation of ribosomal protein S6 (the p70 S6 kinase substrate) (Fig. 7 and Supplementary Fig. 4). Moreover, inactivation of mTORC1 signalling in *Rptor* knockout mice results in microcephaly associated with reduced neural progenitor cell proliferation, slowing of neural progenitor cell cycle, and increased neuroblast apoptosis, as well as proteinuria and progressive glomerulosclerosis (Godel *et al.*, 2011; Cloetta *et al.*, 2013). We attempted to rescue reduced proliferation of NCS fibroblasts with uridine (pyrimidine) supplementation, but did not observe improvements in cell proliferation or morphology (data not shown).

Key functions of WDR73 *in vivo* remain to be defined, but the consistent and severe NCS phenotype reported here and by Colin *et al.* (2014) indicates that WDR73 has specific and indispensable roles in generation, proliferation, and/or viability of cortical neuroblasts, development of the cerebellar granule cell layer, survival of cholinergic striatal interneurons, central visual pathway development, and podocyte biology.

Severe cerebellar pathology in NCS most closely resembles classical descriptions of granular layer aplasia (Smeyne and Goldowitz, 1989; Harding and Copp, 2008). In foetal mice, a transient external granular layer arises by proliferation of cells lining the fourth ventricle (Miale and Sidman, 1961). These cells migrate over the external cerebellar surface and continue to proliferate rapidly until a few weeks after birth. The murine external granular layer disappears during the third postnatal week, as its cells migrate inward past Purkinje neurons to form a compact layer of small granule cell neurons, the internal granular layer (ten Donkelaar *et al.*, 2003). Weaver mice (*Kcnj6* mutation) have selective and profound depletion of cerebellar granule cells caused mainly by accelerated cellular senescence (Smeyne and Goldowitz, 1989; Harkins and Fox, 2002; Takahashi *et al.*, 2006).

In human cerebellum, intensive cell proliferation occurs within the external granular layer between the 28th and 34th gestational weeks and is still active by the fifth postnatal month (Abraham *et al.*, 2001). The mature number of granule cells will exceed 50 billion, accounting for 75% of neurons in the human brain. Hypoplasia and degeneration of cerebellar tissue observed in NCS reflects massive aplasia and/or depletion of this cerebellar granule cell population and its associated neuropil due to reduced proliferation, accelerated demise, or both (Harding and Copp, 2008). This appears to entrain reactive remodelling and loss of Purkinje neurons, which are markedly dysmorphic, with ‘weeping’ dendritic branches, spiked dendritic asteroid bodies, somal sprouts, and bulbous ‘torpedo’ swellings of proximal axons. The presence of somal sprouts on the surface of Purkinje cells was a remarkable finding in both NCS brains. These protrusions are only rarely observed in granule layer aplasia, and have only been reported in Menkes’ disease (OMIM 309400), an X-linked disorder of copper metabolism (Harding, 2013). Although deep cerebellar nuclei remain intact in NCS brain, the histopathology suggests a profound disruption of cerebellar feedback to the nervous system, which must contribute significantly to visual (Voogd *et al.*, 2012; Saglam *et al.*, 2014), sensorimotor (Volpe, 2009; Manto and Oulad Ben Taib, 2013), and cognitive (Barton, 2012; Hoche *et al.*, 2014) disabilities of affected individuals.

Selective depletion of striatal cholinergic interneurons alters signalling through cortico-basal-thalamic circuits to produce an array of extrapyramidal movements (i.e. dystonia, chorea, restlessness) (Pisani *et al.*, 2007; Aosaki *et al.*, 2010; Goldberg *et al.*, 2012). We found further evidence of selective neuronal vulnerability within lateral geniculate nuclei, which were depleted of magno- and parvocellular neurons and devoid of their hexalaminar organization. We have no way to determine if this reflects a paucity of anterograde trophic signalling from the retinae to the lateral geniculate nuclei, or if primary neuronal hypoplasia within the lateral geniculate nuclei or striate cortex leads to transneuronal, retrograde atrophy of retinal ganglion cell axons within the optic tracts. Selective injury to any neuronal population within the central visual system appears to entrain degeneration of all neural elements within the pathway (You *et al.*, 2012; Hendrickson *et al.*, 2013).

Glomerular findings in NCS are those of classic steroid non-responsive focal segmental glomerulosclerosis (D'Agati *et al.*, 2011; D'Agati, 2012). Podocyte injury and depletion is common to all forms of focal segmental glomerulosclerosis (Barisoni, 2012; D'Agati, 2012) and was evident on electron micrographs from NCS kidney, which showed effacement and microvillus remodelling of 80–90% of podocytes. Morphological and functional changes of NCS kidney reflect an important but as yet poorly understood role of WDR73 in podocyte biology (Colin *et al.*, 2014). We observed ‘striped fibrosis’ and tubular atrophy resembling that caused by calcineurin inhibitors, cyclosporine and tacrolimus (Shihab, 1996; Liptak and Ivanyi, 2006). These changes could be non-specific, but may also be a clue to underlying signalling mechanisms relevant to NCS. For example, the mammalian target of rapamycin (mTOR), a key determinant of human cortical growth and organization (Orlova and Crino, 2010; Cloetta *et al.*, 2013; Lim and Crino, 2013; Tsai *et al.*, 2014), also influences podocyte shape and function (Godel *et al.*, 2011; Cina *et al.*, 2012; Jeruschke *et al.*, 2013; Muller-Krebs *et al.*, 2013; Zaza *et al.*, 2014). Recent evidence suggests an important interaction between mTOR and calcineurin pathways in renal cell lines (Basu *et al.*, 2011, 2012), and this could provide new insights into the pathophysiology of NCS.

Although genetic evidence for pathogenicity of *WDR73* c.888delT is definitive, the linked truncating variant in *WHAMM* (c.1264_1270delATAAAAG) (NM_001080435.2) merits additional investigation. As the phenotypical outcome of loss of WHAMM function alone is currently unknown we cannot exclude the possibility that patients doubly homozygous for *WHAMM* and *WDR73* mutations may have a modified phenotype. Further long-term clinical assessments of these individuals, or the identification of subjects who are homozygous for *WHAMM* c.1264_1270delATAAAAG but heterozygous (or wild-type) for *WDR73*, may help to clarify this question. However, our data, corroborated by recent observations of Colin *et al.* (2014), support the assertion that WDR73 loss-of-function is sufficient to cause NCS on the Galloway-Mowat spectrum.

## Abbreviations

NCS: nephrocerebellar syndrome
NHDF: normal human dermal fibroblast
SNP: single nucleotide polymorphism
